# Association of Sarcopenia and Expression of Interleukin-16 in Gastric Cancer Survival

**DOI:** 10.3390/nu14040838

**Published:** 2022-02-17

**Authors:** Jianping Xiong, Haitao Hu, Wenzhe Kang, Xinxin Shao, Yang Li, Peng Jin, Yantao Tian

**Affiliations:** Department of Pancreatic and Gastric Surgery, National Cancer Center/National Clinical Research Center for Cancer/Cancer Hospital, Chinese Academy of Medical Sciences and Peking Union Medical College, Beijing 100730, China; dr_xjp@163.com (J.X.); huhaitao96@163.com (H.H.); kwz@whu.edu.cn (W.K.); shaoxinxin89@163.com (X.S.); bourneliyang@student.pumc.edu.cn (Y.L.); yfywk2014@163.com (P.J.)

**Keywords:** gastric cancer, sarcopenia, inflammation, interleukin-16

## Abstract

We designed the present work to explore the connection between sarcopenia and interleukin-16 (IL-16) expression and their integrated relation with gastric cancer (GC) survival. We deemed the sex-specific third lumbar vertebra skeletal muscle index cutoffs for sarcopenia to be ≤40.8 and ≤34.9 cm^2^/m^2^ in male and female patients, respectively. Immunohistochemistry was carried out to detect IL-16 levels among GC tissues of the patients. We determined overall survival (OS) and relapse-free survival (RFS) by univariate and multivariate analyses. This study included 225 GC cases, with an average age of 62.7 years. There were 41 (18.2%) female patients, and 107 (47.5%) patients had sarcopenia. Sarcopenia and high IL-16 expression were identified as independent factors to predict OS (hazard ratios [HR] = 1.64 and 1.79, 95% confidence interval [CI] = 1.25–2.23 and 1.16–2.78, respectively) and RFS (HR = 1.43 and 1.60, 95% CI = 1.15–2.95 and 1.10–2.37, respectively). There were more cases showing high IL-16 expression detected in the sarcopenia group (55.7% vs. 37.3%, *p* = 0.003). Later, we grouped the patients with sarcopenia and IL-16 expression and discovered that the patients with sarcopenia and IL-16 upregulation displayed the poorest OS (HR = 3.02; 95% CI = 1.64–5.91) and RFS (HR = 2.34; 95% CI = 1.47–4.69). In conclusion, more IL-16 upregulation was noted in GC patients with sarcopenia. Sarcopenia accompanied by high IL-16 expression remarkably indicates a dismal prognosis in GC patients. This suggests that these biomarkers may be able to identify patients with GC with poor prognosis and enhance prognostication.

## 1. Introduction

Gastric cancer (GC) is the fifth most commonly seen cancer, and it is reported to affect an annual number of 1,033,701 cases. In the meantime, GC is the third most significant cause resulting in cancer-associated mortality, which causes 782,685 deaths annually [1]. Sarcopenia is a syndrome with low muscle quality and poor muscle strength that has been frequently seen during the natural aging process or additional health disorders, such as liver failure, liver cirrhosis, cancer, and cognitive disorder [2]. It is suggested that sarcopenia plays an increasingly critical role in cancer because poor muscle strength has been identified as the factor that predicts dismal cancer survival. Sarcopenia is frequently seen among GC patients, with a prevalence of over 7–57.4% in GC patients [3]. It also predicts the dismal survival of GC patients undergoing surgical resection [4]. Some research has been conducted to investigate the mechanism underlying sarcopenia, particularly its relation with inflammation. Inflammation is a critical contributing factor for disease pathology related to the dysfunction of skeletal muscles [5]. Typically, proinflammatory factors inducing anticancer systemic inflammatory responses have been related to additional muscle decomposition and sarcopenia [6]. Interleukin-16 (IL-16), which is a proinflammatory cytokine, plays an important role in chronic inflammatory diseases, such as rheumatoid arthritis and inflammatory bowel disease [7,8]. In addition, IL-16 is associated with the development and progression of malignancy [9,10,11]. Research reports indicate that GC cases showing high serum IL-16 levels have poor survival [12]. However, the association of sarcopenia with IL-16 among GC cases has not been investigated thus far. The combination of IL-16 expression and sarcopenia may be able to identify patients with GC with a poor prognosis. According to the latest Asian Working Group for Sarcopenia (AWGS) guideline, sarcopenia was defined as the combination of low muscle mass plus low grip strength or slow gait speed. Thus, the present work explored the relationship of sarcopenia and IL-16 with GC patient survival.

## 2. Patients and Methods

### 2.1. Study Population

The present retrospective article evaluated GC patients who underwent surgical resection at the Department of Pancreatic and Gastric Surgery, the National Cancer Center/Cancer Hospital, Chinese Academy of Medical Sciences and Peking Union Medical College between June 2011 and July 2016. Patients conforming to the following criteria were enrolled: (1) Patients with histological or cytological diagnosis of GC and receiving preoperative abdominal computed tomography (CT) scan examination in our hospital, (2) patients with a follow-up period of at least 12 months, (3) patients who did not have any other infection or inflammatory disease (no evident sign or symptom of infectious disease, white blood cell WBC count within the normal range), (4) patients who did not have any other metastatic lesion or other primary cancer. Additionally, we also analyzed patients’ laboratory, demographic, and histopathological data and collected related data based on patient records in this institute and relevant databases. The collected data included age, sex, body mass index (BMI, kg/m^2^), Lauren classification, serum albumin, tumor differentiation, tumor size (cm), lymphatic invasion, perineural invasion, vascular invasion, tumor location, survival, and American Joint Committee on Cancer (AJCC 8th) TNM classification.

The present work deemed overall survival (OS) as the duration from surgery to the final follow-up or death due to any cause and relapse-free survival (RFS) as the duration from surgery to death or disease recurrence. We regarded all-cause death as the event. We conducted the final follow-up in March 2020. In the follow-up period, we collected survival data based on telephone interviews or patient medical records. Our study protocols gained approval from the Institutional Review Board of National Cancer Center/Cancer Hospital, Chinese Academy of Medical Sciences and Peking Union Medical College, and each patient signed informed consent prior to enrollment. This work was carried out following the Declaration of Helsinki.

### 2.2. Sarcopenia Definition

According to the latest Asian Working Group for Sarcopenia (AWGS) guideline, sarcopenia was defined as the combination of low muscle mass plus low grip strength or slow gait speed [13]. Because the design of our study is retrospective, information about muscle function (muscle strength or physical performance) cannot be collected. Thus, we focused on muscle mass evaluation to determine sarcopenic patients. By adopting the public semiautomatic tool (body mass index (BMI) measurement tools, version 1.0 (https://sourceforge.net/projects/muscle-fat-area-measurement accessed on 12 November 2021), the −29 to 150 Hounsfield units were set as the threshold at the third lumbar vertebra (L3) level to measure the cross-sectional areas (CSA) of oblique/transverse abdominal muscles, paraspinal/psoas muscles, and rectal [14]. By adopting the deidentified Digital Imaging and Communications in Medicine files, a radiologist who was blinded to patient data and had five years of related experience was invited for analysis. Later, we normalized the L3 skeletal muscle index (SMI) based on patient stature through the division of muscle area by squared height, namely, total lumbar muscle CSA (cm^2^)/height (m^2^). In this study, we deemed the sex-specific L3 SMI threshold for sarcopenia to be ≤40.8 and ≤34.9 cm^2^/m^2^ for male and female patients, respectively, which was created based on Zhuang et al. for the Chinese population [15].

### 2.3. IL-16 Level Detected by Immunohistochemistry (IHC)

Anti-IL-16 primary antibodies were utilized to perform IHC staining on tissues. For immunostaining staining, the specimens were cut into 4–6 μm thick sections, deparaffinized, and rehydrated, and 3% hydrogen peroxide in methanol was used for the blockage of endogenous peroxidase at RT. Then, the sections were washed with phosphate-buffered saline (PBS; pH 7.2–7.6, three times), and the sections were washed with PBS again after heat-mediated antigen retrieval was performed. Subsequently, the sections were incubated with primary antibodies overnight at 4 °C with primary antibody against IL-16. Then, sections were washed with PBS and incubated with the respective secondary antibody (kit-5020, Maixin, china). The slides were rinsed in PBS again, treated with diaminobenzidine (DAB; 1:50) for 1–3 min, and finally counterstained with hematoxylin, according to a standard protocol. Images were acquired on a Leica DM500 microscope (Leica Biosystems, Germany). Later, two pathologists were invited to analyze the staining results. IL-16 levels within GC samples were evaluated by the IHC score approach. Notably, we rated IHC scores according to staining intensity as well as positive cell proportion. Specifically, we divided intensity scores into 0, 1, 2, and 3, which represented no, weak, moderate, and strong staining, respectively. In addition, positive cell proportions were also divided into 0, 1, 2, 3, and 4, which represented <5%, 6–25%, 26–50%, 51–75%, and >75%, respectively. Then, we multiplied intensity with proportion scores to obtain the final result, which was 0–12. Later, we chose 6 as the threshold for dividing high (≥6) or low (<6) expression [16,17] (Figure S1).

### 2.4. Statistical Analysis

Chi-square tests and t-tests were used to analyze categorical and continuous data, respectively. Thereafter, we plotted the Kaplan–Meier (K-M) survival curve and examined heterogeneities in curves by log-rank test. Hazard ratios (HRs) were calculated by multivariate Cox regression. *p* < 0.05 (two-sided) stood for statistical significance. Statistical analyses were completed using Rver. 4.0.2 (R Foundation for Statistical Computing, Vienna, Austria), SPSS 18.0 (SPSS Inc., Chicago, IL, USA), and GraphPad Prism 7 software (GraphPad Software, San Diego, CA, USA).

## 3. Results

### 3.1. Clinicopathological Characteristics of Patients

We classified patients into two groups based on whether they had sarcopenia. Table 1 presents the clinicopathological features of all patients, which included sex, age, BMI (kg/m^2^), ASA score, tumor size (cm), Lauren classification, serum albumin, lymphatic invasion, perineural invasion, vascular invasion, tumor location, and TNM stage. This work enrolled 225 GC patients, with an average age of 62.7 years. There were 41 (18.2%) female patients. According to the thresholds, 107 (47.5%) cases had sarcopenia. In addition, sarcopenia was associated with advanced age (≥65.0 years), TNM stage, and decreased BMI (<18.5 kg/m^2^).

### 3.2. Sarcopenia and Prognosis

**Figure 1:**
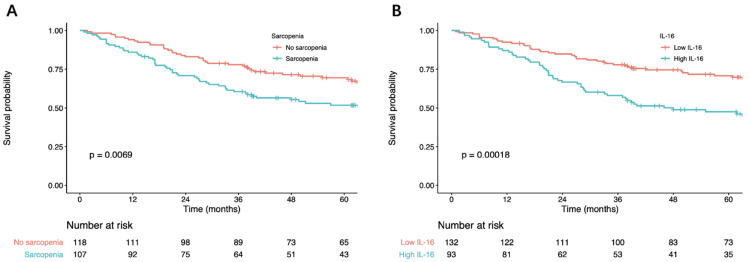
(**A**) Overall survival of gastric cancer patients with and without sarcopenia. (**B**) Overall survival of gastric cancer patients with low and high IL-16. IL-16, interleukin-16.

This study also compared the OS and RFS of GC patients between sarcopenia and nonsarcopenia groups. Kaplan–Meier curves showed a significant difference in OS between the sarcopenia and nonsarcopenia groups (*p* < 0.001 upon log-rank test) (Figure 1A). Multivariate Cox regression survival analysis also showed inferior OS (HR = 1.43; 95% CI = 1.15–2.95, *p* < 0.001) and RFS (HR = 1.64; 95% CI = 1.25–2.23, *p* < 0.001) in patients with sarcopenia (Table 2).

### 3.3. IL-6 and Survival

There were 93 (41.4%) patients with high IL-16 expression, and they were associated with poor survival (Figure 1B). We discovered that IL-16 could independently predict OS (HR = 1.60; 95% CI = 1.10–2.37, *p* < 0.001) and RFS (HR = 1.79; 95% CI = 1.16–2.78, *p* < 0.001) upon multivariate analysis (Table 2).

### 3.4. Sarcopenia and IL-6

**Figure 2:**
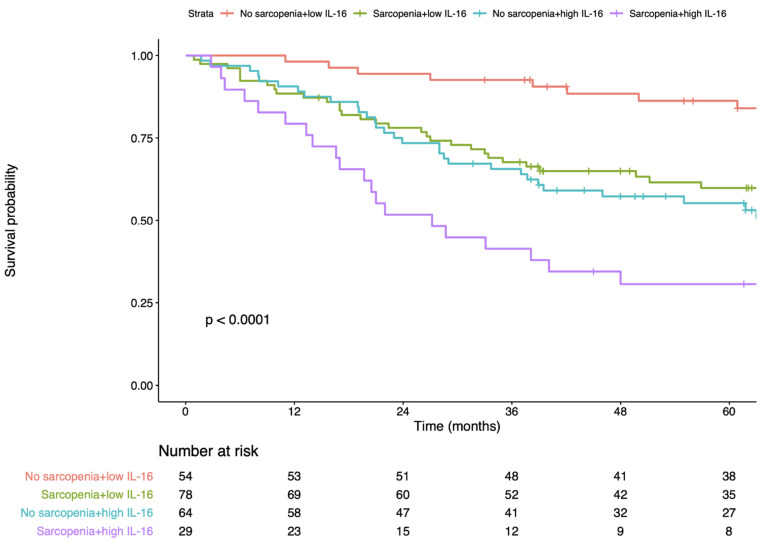
Kaplan–Meier plots of overall survival in gastric cancer. A significant difference was noted between the patients’ groups stratified by sarcopenia status and IL-16 expression in overall survival. IL-16, interleukin-16.

The sarcopenia group had more cases showing IL-16 upregulation (55.7% vs. 37.3%, *p* = 0.003) (Table 1). Later, we combined sarcopenia with IL-16 levels to analyze their relationship with prognosis. As shown in the Kaplan–Meier curves, sarcopenia cases showing IL-16 upregulation displayed the poorest prognosis, while patients without sarcopenia and IL-16 downregulation displayed the most favorable survival (*p* < 0.001 upon log-rank test) (Figure 2). Patients with both sarcopenia and high IL-16 expression had significantly poorer OS (HR, 2.43; 95% CI, 1.47–4.69) and RFS (HR, 3.02; 95% CI, 1.64–5.91) than those without any of the above two risk factors. When sarcopenia was accompanied by high IL-16 expression and GC survival stratified by age, sex, body mass index, and pTNM stage, they had markedly impaired survival stratified by age, sex, BMI, and pTNM stage. pTNM stage I and age <65 years in patients with sarcopenia and high IL-16 expression also had markedly impaired survival compared with age- and stage-matched patients without any of the above factors (Table 3).

## 4. Discussion

This study is the first to examine and report the association of sarcopenia with IL-16 levels in GC cases, as well as their integrated relation with GC survival. Based on our results, higher IL-16 expression was detected in GC patients with sarcopenia. Sarcopenia and high IL-16 expression predicted the dismal prognosis of GC cases.

Inflammation is hypothesized to underlie and be aggravated through muscle decomposition, which is involved in the mutually reinforcing cycle that facilitates tumor development. TNF-β, IL-6, IL-8, IL-12, IL-23, and C reactive protein (CRP) are related to muscle mass loss. TGF-β is suggested to trigger fibrosis and atrophy of skeletal muscles by modulating skeletal muscle satellite cell activity. Sarcopenia has been found to be markedly related to elevated TGF-β expression in different processes, such as muscle injury, fibrosis, and regeneration. IL-6, one of the glycoproteins, is constituted by 184 amino acids and contains four long a-helices, which are generated via innate immune cells, such as fibroblasts, mesenchymal cells, monocytes, and macrophages [18,19]. As revealed by Reisinger et al., the poor muscle mass among cases receiving surgical resection for CRC was not related to the plasma concentrations of IL-6 [20]. However, Rong et al. showed that IL-6 is associated with muscle mass loss among elderly male and female populations [21]. As suggested by the European Society for Parenteral and Enteral Nutrition expert group, IL-6 can reduce muscle mass [22]. IL-12 represents one of the proinflammatory factors promoting the growth and cytotoxicity of T cells [23]. Romanazzo et al. identified that IL-12 enhanced the C2C12 skeletal muscle cells of mice to differentiate into myogenic lineages [24]. As discovered in recent research, decreased IL-12 expression is related to increased sarcopenia incidence [25]. Increased serum IL-8 levels are associated with cachexia and sarcopenia in pancreatic cancer [26]. Hu et al. reported that IL-23 upregulation within tissues was related to poor SMI, and the combination of these two predicted the dismal survival of CRC cases [27]. This study was the first to investigate the relationship of IL-16 upregulation within tissues with low SMI, as well as their pooled relation with GC survival.

Prior works also discovered that the neutrophil-to-lymphocyte ratio (NLR) and C-reactive protein (CRP) are important factors leading to muscle decomposition among tumor cases. CRP is a clinical indicator that is often used to assess the inflammatory state of the body. Its levels rise significantly when the body is infected or damaged. A meta-analysis including 3072 sarcopenia cases and 8177 controls indicated that CRP levels were significantly elevated in people with sarcopenia [28]. Sarcopenia and the increased modified Glasgow prognostic score (mGPS) containing CRP were independently related to poor prognosis of cases showing local renal cell carcinoma (RCC) [29]. Additionally, colorectal cancer (CRC) cases with increased NLRs are associated with a markedly decreased skeletal muscle index, whereas CRC patients who have sarcopenia and increased NLRs exhibit poor OS rates [30]. For small cell lung cancer (SCLC) cases, the presence of sarcopenia and increased NLRs predict poor prognosis [31]. Meanwhile, the presence of sarcopenia combined with NLRs in the first diagnosis predicts markedly dismal OS and RFS among patients undergoing chemoradiotherapy due to local head and neck cancer (HNC) [32]. In the presence of sarcopenia, NLR > 3 significantly predicted dismal OS and PFS in advanced biliary tract cancer [33]. Shigeto et al. showed that “an increased NLR and muscle atrophy” represented a factor to predicting the prognosis of stage IV GC [34].

There are certain limitations in the present work. First, we retrospectively collected data from a single center, which was associated with unavoidable selection bias, although we screened samples in strict accordance with our preset criteria. Because the nature of our study is retrospective, we cannot obtain the blood plasma of patients to further study the relationship between serum levels of IL-16 and sarcopenia. We initially tried to use Western blotting an RT-qPCR to quantify IL-16 levels in gastric cancer patients without success. We suspect that the reason is that we need to study the effect of IL-16 level on the long-term survival outcome of patients with gastric cancer at the same time, using tumor paraffin tissue that has been preserved for a long time. In the future, prospective articles will be designed to explore the relationship between IL-16 levels and sarcopenia in patients with gastric cancer. Second, the AWGS suggests using the presence of loss of muscle mass plus low muscle function (strength or performance) to define sarcopenia. Because of the retrospective nature of our study, information about muscle function (muscle strength or physical performance) cannot be collected. Thus, we focused on muscle mass evaluation to determine sarcopenic patients. The present work selected the definition of sarcopenia proposed by Zhuang et al., which defined sarcopenia standards for the Chinese population [35]. Typically, the threshold L3-SMI values to diagnose sarcopenia were deemed 40.8 and 34.9 cm^2^/m^2^ for males and females, respectively. Our findings had poor generalizability to Western populations because the thresholds of the L3 SMI adopted in the present work were geographic-region-specific. Third, this study excluded patients receiving neoadjuvant chemotherapy (NACT), and the results were not applicable to GC patients postNACT.

## 5. Conclusions

In conclusion, the present work is the first to investigate the close relationship of sarcopenia with IL-16 in GC. The presence of sarcopenia accompanied by the inflammatory factor IL-16 remarkably indicates a dismal prognosis. More prospective studies are warranted to validate the results in this work and identify more biomarkers.

## Figures and Tables

**Table 1:** Baseline clinicopathologic characteristics. BMI, body mass index. SMI, skeletal muscle index.

Clinicopathological Features	All Cases	Sarcopenia	Non-Sarcopenia	*p* Value
	(*n* = 225)	(*n* = 107)	(*n* = 118)	
Age				0.006
<65.0	537 (66.2)	170 (44.6)	269 (62.5)	
≥65.0	275 (43.8)	212 (55.4)	161 (37.5)	
Gender				0.726
Male	184 (81.8)	88 (82.3)	96 (81.3)	
Female	41 (18.2)	19 (17.7)	22 (18.7)	
BMI (kg/m^2^)				<0.001
<18.5	16 (7.1)	13 (12.5)	3 (2.5)	
≥18.5	209 (92.9)	94 (87.5)	115 (97.5)	
ASA score				0.565
1	17 (7.6)	7 (6.5)	10 (8.5)	
2	183 (81.4)	87 (81.3)	96 (81.3)	
3	25 (11.0)	13 (12.2)	12 (10.2)	
Tumor size (cm, median)	4.3	4.6	4.4	0.16
Vascular invasion				0.731
Negative	140 (62.4)	65 (60.7)	75 (63.7)	
Positive	85 (37.6)	42 (39.3)	43 (36.3)	
Perineural invasion				0.453
Negative	123 (54.8)	56 (52.1)	67 (57.3)	
Positive	102 (45.2)	51 (47.9)	51 (42.4)	
Tumor location				0.282
Upper	62 (27.5)	27 (25.6)	35 (29.5)	
Middle/Lower	163 (72.5)	80 (74.4)	83 (70.5)	
Lauren Classification				0.101
Intestinal-type	89 (39.5)	38 (35.8)	51 (43.2)	
Diffused-type	77 (34.3)	39 (36.9)	38 (32.2)	
Mixed	59 (26.2)	30 (28.3)	29 (24.6)	
Serum albumin (g/dL)				0.02
≥3.5	163 (72.6)	71 (66.3)	92 (78.2)	
<3.5	62 (27.4)	36 (33.6)	26 (21.8)	
pTNM stage				0.019
I	47 (20.9)	14 (13.1)	33 (27.9)	
II	61 (27.1)	31(29.0)	30 (25.4)	
III	117 (52.0)	62 (57.9)	45 (38.1)	
L3 SMI (cm^2^/m^2^), median	38.8	32.3	44.2	<0.001
Interleukin-16 expression				0.003
Low	132 (58.6)	48 (44.3)	74 (62.7)	
high	93 (41.4)	59 (55.7)	44 (37.3)	

**Table 2:** Sarcopenia, interleukin-16, and gastric cancer survival. Cox proportional hazards models adjusted for age, gender, BMI, tumor size, tumor differentiation, Lauren classification, lymphatic invasion, vascular invasion, perineural invasion, tumor location, pTNM stage. BMI, Body Mass Index.

Variables	HR (95% CI)	
	Overall Survival	Relapse-Free Survival
Sarcopenia Without With	1.64 (1.25, 2.23)	Reference 1.43 (1.15, 2.95)
Interleukin-16 expression low high	Reference 1.79 (1.16, 2.78)	Reference 1.60 (1.10, 2.37)
High interleukin-16 expression and sarcopenia Neither Both	Reference 3.02 (1.64, 5.91)	Reference 2.34 (1.47, 4.69)

**Table 3:** Sarcopenia accompanied by high interleukin-16 expression and gastric cancer survival stratified by age, gender, body mass index, and pTNM stage. BMI, Body Mass Index. Cox proportional hazards models adjust for age, gender, BMI, tumor size, tumor differentiation, Lauren classification, lymphatic invasion, vascular invasion, perineural invasion, tumor location, pTNM stage unless stratified by those variables.

Stratification Variable	HR (95% CI)	
	Overall Survival	Relapse-Free Survival
Age		
<65.0	2.37 (1.48, 7.04)	1.82 (1.25, 5.73)
≥65.0	3.41 (1.55, 7.23)	2.76 (1.40, 5.91)
Gender		
Male	2.83 (1.50, 6.37)	2.25 (1.31, 5.08)
Female	3.19 (1.61, 7.14)	2.59 (1.41, 5.95)
BMI (kg/m^2^)		
<18.5	2.12 (0.81, 8.75)	1.63 (0.76, 4.42)
≥18.5	3.38 (1.45, 7.90)	2.94 (1.26, 5.71)
pTNM stage		
I	1.65 (1.17, 9.82)	1.48 (1.12, 6.19)
II	2.79 (1.35, 8.73)	2.06 (1.34, 5.07)
III	3.47 (2.31, 8.41)	2.72 (1.24, 5.21)

## Data Availability

Not applicable.

## Supplementary Materials

The following are available online at https://www.mdpi.com/article/10.3390/nu14040838/s1, Figure S1. Representative samples of high IL-16 expression in gastric cancer.
