# Supplementary figures and images for: Association of Sarcopenia and Expression of Interleukin-16 in Gastric Cancer Survival
Source: Nutrients. 2022 Feb 17;14(4):838. doi: 10.3390/nu14040838 (PMC8878671; doi:10.3390/nu14040838)

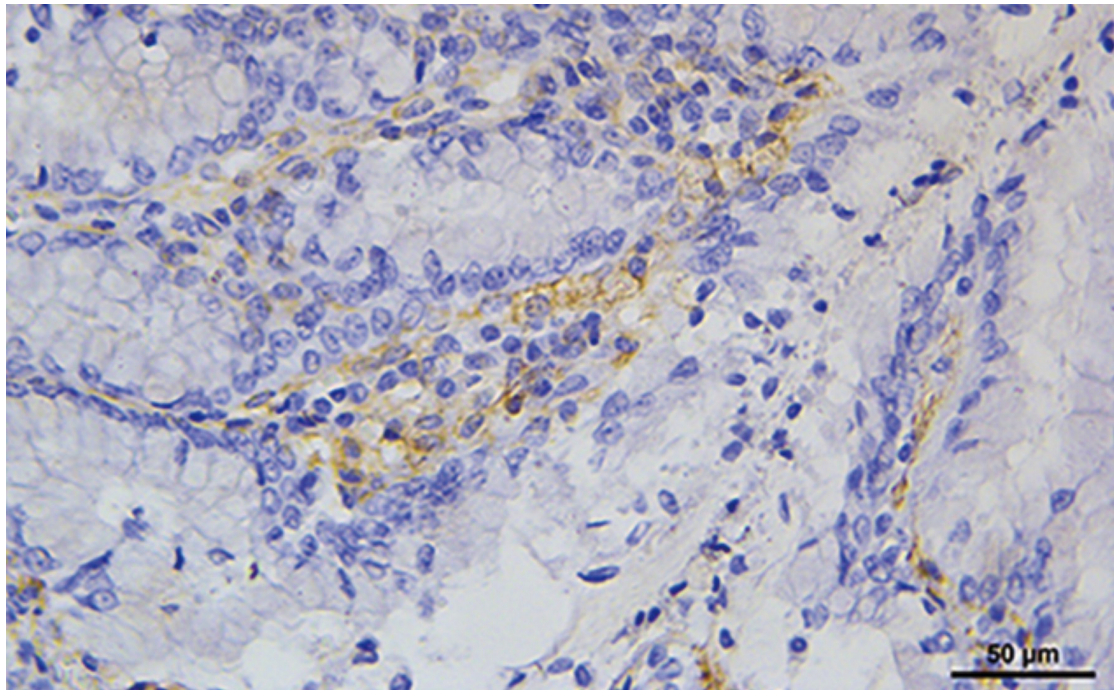

Figure. S1. Representative samples of high IL-16 expression in gastric cancer.

Supplement: Supplementary file 1 [file nutrients-14-00838-s001.zip › nutrients-1555927-supplementary.pdf]
